# Chameleon-Inspired Mechanochromic Photonic Elastomer with Brilliant Structural Color and Stable Optical Response for Human Motion Visualization

**DOI:** 10.3390/polym15122635

**Published:** 2023-06-09

**Authors:** Yanbo Zhao, Kai Zhao, Zhumin Yu, Changqing Ye

**Affiliations:** School of Materials Science and Engineering, Suzhou University of Science and Technology, Suzhou 215009, China; zyb1763001288@163.com (Y.Z.); yuzhumin1998@163.com (Z.Y.)

**Keywords:** photonic elastomer, structural color, mechanochromism, visualized sensing, sandwich structure

## Abstract

Flexible and stretchable electronic devices are indispensable parts of wearable devices. However, these electronics employ electrical transducing modes and lack the ability to visually respond to external stimuli, restricting their versatile application in the visualized human–machine interaction. Inspired by the color variation of chameleons’ skin, we developed a series of novel mechanochromic photonic elastomers (PEs) with brilliant structural colors and a stable optical response. Typically, these PEs with a sandwich structure were prepared by embedding PS@SiO_2_ photonic crystals (PCs)within the polydimethylsiloxane (PDMS) elastomer. Benefiting from this structure, these PEs exhibit not only bright structural colors, but also superior structural integrity. Notably, they possess excellent mechanochromism through lattice spacing regulation, and their optical responses are stably maintained even when suffering from 100 stretching–releasing cycles, showing superior stability and reliability and excellent durability. Moreover, a variety of patterned PEs were successfully obtained through a facile mask method, which provides great inspiration to create intelligent patterns and displays. Based on these merits, such PEs can be utilized as visualized wearable devices for detecting various human joint movements in real time. This work offers a new strategy for realizing visualized interactions based on PEs, showing huge application prospects in photonic skins, soft robotics, and human–machine interactions.

## 1. Introduction

With the rapid development of wearable technology, a variety of wearable electronic devices are emerging accordingly. Among the different types of devices, flexible and stretchable electronic devices have attracted great attention because they can convert deformation induced by external mechanical stimuli into measurable electrical signals [1,2,3,4,5]. Based on this, such devices are widely used to detect the movement of human joints and physiological signals (such as the pulse, heartbeat, etc.), which has become a hot topic in scientific research. To date, great efforts have been dedicated to the development of different types of flexible electronic materials. In general, they are obtained by incorporating conductive sensing materials (metal nanoparticles, graphene, carbon nanotubes, graphite, and so on) with an elastic polymer matrix (such as silicon rubber, polyurethane, and polyurea) [6,7]. Through the optimization of the device structure or the development of new sensing materials, these devices have been designed to possess high sensitivity, rapid responses and excellent stability of the electrical responses. However, these electronics almost adopt electrical signals to feedback information [8,9], which needs to be further processed by connecting additional electronic devices for data presentation [10,11]. In addition, the outputted electrical signals cannot be observed intuitively by the naked eye, which limits their application in visualized human–machine interactions [12,13]. Furthermore, such electrical signals will inevitably be affected by various interferences (e.g., temperature and humidity) during practical uses, which will lead to a decrease in the signal-to-noise ratio and an inability to accurately detect the real health status of the human body [14,15]. To this end, flexible visualized devices that can directly convert external mechanical and physical stimuli into eye-perceptible color changes would be of great benefit to significantly simplify the structures of these devices and will be an effective strategy to solve the above problems [16,17].

Photonic crystals (PCs), which are periodic arrays composed of dielectric materials with different refractive indices, are typically characterized by their photonic band gap (PBG) [18,19]. When the PBG is located within the visible range, PCs can selectively reflect light with specific wavelengths and show bright structural colors as a result [20]. The PBG position of photonic crystals follows Bragg’s law and depends on the effective refractive index (RI) and the periodic structure. Intriguingly, many organisms in nature possess vivid and dynamic structural color patterns. such as the abundant colors of butterfly wings, peacock feathers, and chameleon skin. Among them, the most typical representatives are chameleons. To be specific, they can change their skin color by actively adjusting the lattice spacing of guanine nanocrystal arrays within iridophore cells to adapt to the external environment [21,22,23]. This unique structural color variation imparts their ability to adapt to the environment stimuli. Inspired by this fascinating phenomenon, a series of stimuli-responsive PCs have been designed and emerged in recent years [24,25,26,27]. Among them, the mechanically-responsive PCs can output the color variation through the lattice spacing regulation under external force, thereby allowing visual feedback for invisible mechanical stress [28]. Typically, they are prepared by incorporating PCs with elastic matrix, such as hydrogels [29,30] or polymeric elastomers [16,31]. Hydrogels as a class of smart materials are widely studied for their excellent flexibility, elastic mechanics, and sensitive responses. Although photonic hydrogels possess sensitive mechanochromism ability and superior biocompatibility, they are limited by their low mechanical strength and environmental instability induced by their high water content [17,32]. Fortunately, photonic elastomers (PEs) demonstrate good color-changing ability, environmental stability and mechanical properties, which can help solve the above problems [33]. With these merits, they can convert the mechanical deformation into intuitive and steady optical signals. When utilized for motion detection, the real-time motion states of the human body can be instantaneously and intuitively observed by the naked eye without additional electrical equipment, which would be of immense benefit to significantly simplify the device structures. Therefore, this type of PE has good applications in various visualized fields, such as in photonic skin [23,25], human–machine interactions [17,34], health monitoring [15,35,36,37], and soft robotics [38,39].

**Figure 1:**
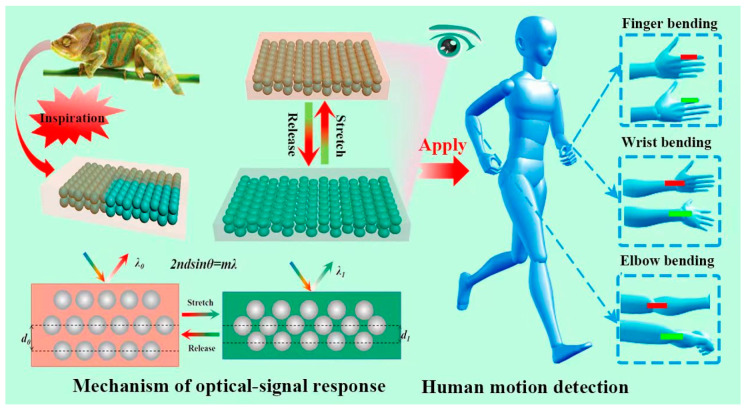
Schematic illustration showing the structure of PEs and their application in human motion detection. Schematics of the chameleon. Reproduced with permission from [25], Wiley-VCH 2022.

Inspired by the color-changing ability of chameleons, we designed and fabricated a series of novel mechanochromic PEs with brilliant structural colors and stable optical responses for visualizing various human motions. As shown in Figure 1, the PEs were prepared by sandwiching PS@SiO_2_ PCs (n = 1.56) within the polydimethylsiloxane (PDMS, n = 1.41) elastomer. Although there are some PDMS based photonic composite films reported, they mainly focus on the improving the purity and saturation of structural colors, as well as the reversible switch from the transparent state to opaqueness. As we all know, the strategy for fabricating brilliant PEs must meet the following two requirements: (1) a large refractive index (RI) difference (>0.1) between the nanospheres and the elastomer, providing the brilliant structural color of the PE; (2) the nanospheres must properly interact with the elastomer, which is critical to the mechanochromic capability of a PE. Benefiting from the large refractive index (RI) difference between PS@SiO_2_ and PDMS (Δn = 0.15 > 0.1) and the sandwiched structure, these resulting films show bright structural colors, high flexibility, superior structural integrity, and stability. Furthermore, the PS@SiO_2_ core-shell nanospheres allow proper interaction with the elastomer because of the formation of a chemical interaction between the PDMS network and hydroxyl group on the surface of PS@SiO_2_ nanospheres, which is critical to the mechanochromic capability of PE. More importantly, the PEs exhibit distinct structural color variation upon stretching via lattice spacing regulation, allowing the visualization of external forces. We also demonstrate that these natural PEs can not only directly display external stimuli in real time but can also quantify the stimuli in a simple fashion. In addition, different patterned PEs were readily prepared using the mask method, which shows the feasibility of smart PC patterns. Based on the above advantages, we further demonstrate their ability as visualized devices for tracking human joint movements, revealing great potential in human–machine interactions, intelligent wearable devices, soft robotics, and so on.

## 2. Materials and Methods

### 2.1. Materials

The sodium dodecyl benzene sulfonate, styrene (St), and vinyltrimethoxysilane (VTMS) were purchased from Aladdin Biochemical Technology Co., Ltd. (Shanghai, China). The potassium peroxydisulfate was provided by Damao Chemical Reagent Factory (Tianjin, China). The PDMS was purchased from Dow Corning Co., Ltd. (Midland, MI, USA). The ammonia (25 wt%) and anhydrous ethanol (AR) were obtained from Fuyu Chemical Reagent Company (Shanghai, China). The St was washed three times using a 10% aqueous hydroxide solution to remove the polymerization inhibitors, and then washed with deionized water until reaching neutrality. The deionized water and high-purity nitrogen (99.999%) were produced by Suzhou University of Science and Technology and Suzhou Wuzhong Jinhong Gas Co., Ltd. (Suzhou, China), respectively. All reagents were used as received without further purification. 

### 2.2. Preparation of PS and PS@SiO_2_ Microspheres

The PS microspheres were prepared by emulsion polymerization [40,41]. The general preparation process was as follows. Firstly, 135 mL of deionized water, an appropriate amount of sodium dodecyl benzene sulfonate, and 10.0 g of the St were put into a 250 mL three-neck flask equipped with a stirrer and a thermometer. Then, the temperature of the above system was heated up to 85 °C through a water bath under the protection of a nitrogen atmosphere. Next, 0.100 g of potassium peroxydisulfate was added until the temperature was stable. Finally, the PS microsphere emulsion was obtained after a 5 h reaction.

The PS@SiO_2_ core–shell microspheres were fabricated using a modified Stöber method [42,43]. Firstly, 5 mL of PS emulsion was dispersed into 57 mL of deionized water and placed on a magnetic stirring plate. Then, 4 mL of ammonia solution and 27 mL of deionized water were added into the above system with stirring. Subsequently, an appropriate amount of VTMS (0.25 to 1 mL) was added dropwise into the reactor with a pipette gun (the amount of VTMS added ultimately determines the particle size of the microsphere). After the drop process was finished, the hydrolysis reaction was proceeded at room temperature for 6 h under continuous mechanically stirring. Finally, the suspension was centrifuged (9500 rpm, 10 min) and rinsed with anhydrous ethanol three times, then the obtained PS@SiO_2_ microspheres were dispersed in ethanol for further use.

### 2.3. Preparation of PEs with a Sandwich Structure

Firstly, the precursor and curing agent of the PDMS were mixed thoroughly (m/m = 10/1) and poured onto the substrate. After degassing, the PDMS precursor was cured on a heating plate for 30 min at 85 °C to form a transparent film. Then, the cured PDMS was treated with oxygen plasma for 2 min. The PS@SiO_2_ dispersion was subsequently coated onto the hydrophilic PDMS on a heating plate at 80 °C. After the dispersion solution was dried, the microspheres self-assembled into a three-dimensional ordered PC structure with a large area within several minutes. Next, the precursor of the PDMS (according to the volume ratio of the precursor and curing agent mentioned above) was infiltrated into the void of the PS@SiO_2_ using strong capillary force to replace air, during which the structural color was red-shifted due to the increase in the effective refractive index. The color shift of the PS@SiO_2_ colloidal crystals indicated that the precursor had completely filled the gaps between them. Finally, the PE (i.e., PDMS/PS@SiO_2_/PDMS composite film) with a sandwich structure was obtained after curing at 85 °C, followed by peeling off from the glass substrate.

### 2.4. Characterization

All SEM images including the morphologies of PS and PS@SiO_2_ microspheres and the cross sections of the PEs were obtained using a scanning electron microscope (Hitachi, SU8020, Tokyo, Japan). Au was sputtered on the surfaces of the samples before tests. The reflectance spectra were measured using a PG2000-Pro fiber optic spectrometer (Shanghai Idea optics Co., Ltd., Shanghai, China). The reflection spectra of the PEs in the initial and stretching states were measured with a fixed incidence angle of 0°. The particle sizes and Zeta potentials of the PS and PS@SiO_2_ microspheres were obtained using a Malvern particle size analyzer (Shanghai Musen Biotechnology Co., Ltd., Shanghai, China). Digital photos of all the samples were taken using a smartphone in natural light. A mechanical tensile test was carried out using a universe testing machine (PT-305, Dongguan Precise Test Equipment Co., Ltd., Dongguan, China). The tensile rate was 5 mm min^− 1^, and the tested samples of PE with a length of 21 mm, a width of 10 mm, and a thickness of 1.5 mm were prepared in a mold.

## 3. Results and Discussion

### 3.1. Preparation and Characterization of PEs

**Figure 2:**
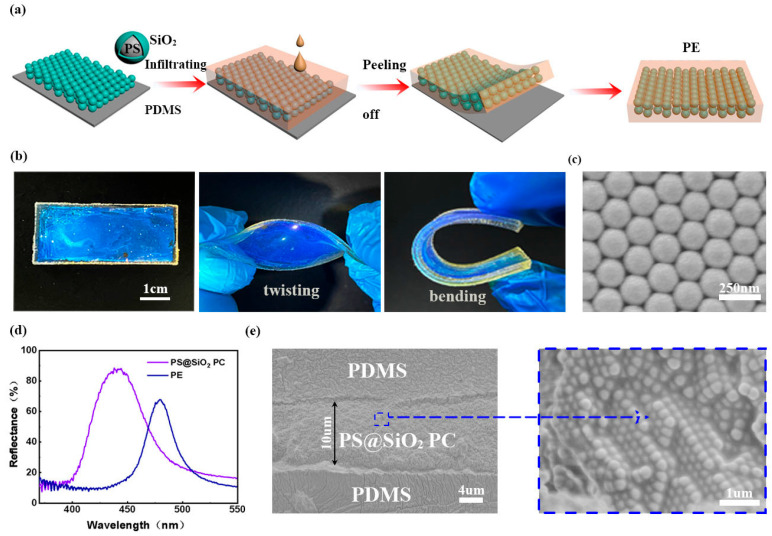
(**a**) Schematic illustration showing the fabrication process of the PE films. (**b**) Photographs of the PE, as well as its twisted and bent states, respectively. (**c**) SEM image of the PS@SiO_2_ PC template. (**d**) Reflectance spectra of the PS@SiO_2_ opal PC and PE, respectively. (**e**) The cross-sectional SEM image of the PE showing its sandwich structure.

As depicted in Figure 2a, the PS@SiO_2_ PC was firstly self-assembled on the hydrophilic-treated PDMS substrate, and its voids were then infiltrated by an additional precursor of PDMS. After curing, the PE with a sandwich structure (PDMS/PS@SiO_2_/PDMS composite film) was successfully prepared (see Section 2, Materials and Methods for details). Due to the excellent mechanical properties of PDMS [44,45], the resultant PE exhibited excellent flexibility and mechanical compliance when subjected to typical deformations, including twisting, bending and stretching (discussed below), making this an ideal candidate for wearable electronics (Figure 2b). Obviously, the highly ordered assemblies and uniform sizes of the PS@SiO_2_ PC and corresponding PS PCs, which are close-packed face-centered-cubic (FCC) structures, can be clearly seen in Figure 2c and Figure S1. As shown in Figure 2d, when the PDMS precursor infiltrated into the voids of the PS@SiO_2_, the reflection wavelength peak of the PC red-shifted from 438 to 479 nm, which displayed a color transition from violet to blue. This was because air (*n*_air_ = 1) in the voids of the PC was replaced by PDMS (*n*_PDMS_ = 1.41), which increased the effective refractive index. According to Bragg’s law [46], the reflection position of the PS@SiO_2_ PC can be theoretically calculated, which is given as follows:

(1) $$\lambda =1.633D{\left(n_{\mathrm{eff}}^{2}-\sin^{2}\theta \right)}^{1/2}$$

(2) $$n_{\mathrm{eff}}^{2}=n_{1}^{2}\phi +n_{2}^{2}\left(1-\phi \right)$$

where *λ* is the maximum reflection wavelength, *D* is the diameter of spheres, *n*_eff_ is the effective refractive index of the PE, *θ* is the angle between the incident light and the reflective surface, *ϕ* is the volume fraction of the particles, *n*_1_ is the refractive index of the particles, and *n*_2_ is the refractive index of the matrix. As the PDMS precursor infiltrates into the voids, the *n*_eff_ increases accordingly, resulting in the red-shift of the *λ*. In addition, the cross-sectional SEM image of the PE in Figure 2e clearly shows its sandwich structure; that is, the PS@SiO_2_ PC was sandwiched between two layers of PDMS. The enlarged SEM image of the PE indicates that the air in the gap between PS@SiO_2_ PCs spheres was completely filled with PDMS, and the PC assemblies transformed into a loosely packed hexagonal structure, which facilitates mechanochromism [24,47].

### 3.2. Mechanochromic Properties of the Pes

**Figure 3:**
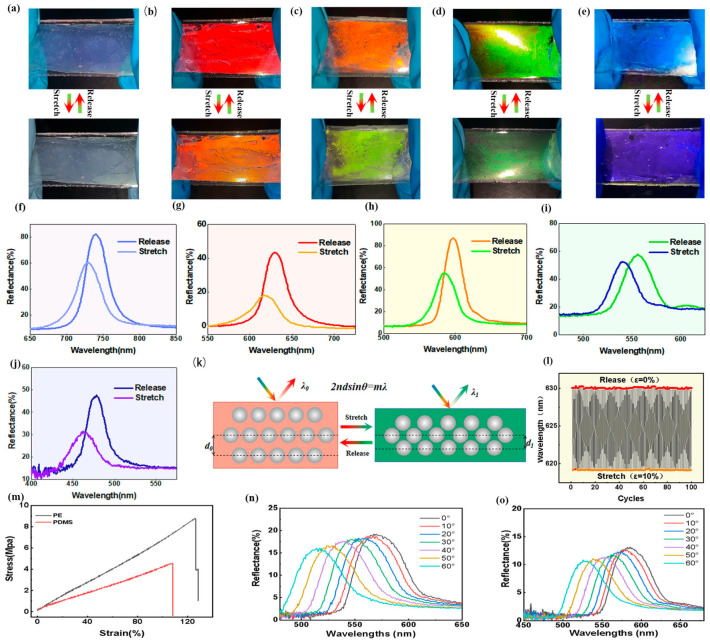
Photographs showing the mechanochromism of the PEs with different structural colors. (**a**) infrared; (**b**) red; (**c**) orange; (**d**) green; (**e**) blue; (**f**–**j**) Reflectance spectra of PEs with different colors during stretching with 10% strain. (**k**) Schematic illustration showing the mechanochromic mechanism of the PEs. (**l**) The reversible changes of the reflection wavelength during 100 stretching–releasing cycles at 0% to 10% strain. (**m**) Typical stress–strain curves of PE and PDMS at a tensile strain rate of 5 mm min^−1^. (**n**–**o**) Reflectance spectra of yellow PS@SiO_2_ and orange PE under different angles.

Notably, the PEs demonstrated superior mechanochromic performance and good reversibility. A series of PEs with different structural colors were prepared by using PS@SiO_2_ PCs with different particle sizes, all of which showed good mechanochromic performance, as can be seen in Figure 3a–e (see the detailed analysis in Figures S2–S9 and the Supporting Information).

Taking the orange-colored PE as an example, its structural color varied gradually from orange to green when stretched from the initial state to 10% strain (Figure 3c). To quantitively evaluate the mechanochromic properties of PEs, optical spectroscopy was also carried out. Obviously, the reflection peak of the orange-colored PE gradually blueshifted from 599 to 583 nm accordingly, further confirming the above color-changing process (Figure 3h). Similarly, the change in structural color and the blue-shift in the wavelength peak can also be observed intuitively in other PEs (i.e., infrared, red, green, and blue PEs) (Figure 3f,g,i,j). The PE demonstrated good mechanochromic properties and exhibits excellent reversibility. The structural color of the orange PE underwent a gradual change from orange to green when longitudinally stretched from 0% to 10%, as shown in Figure 3c. During its elongation from 0% to 20%, the reflection peak gradually shifted from 600 to 583 nm (Figure S10a). As shown in Figure S10b, there was a monotonic decrease in *λ*_max_ with strain up to 20%. Obviously, when the strain ε_x_ < 7.5 %, the *λ*_max_ changes rapidly with increasing strain because the interparticle distance is sufficient to avoid contact between the two nanospheres; the deformation of the PE is dominated by the PDMS elastomer for small strain values. When further increasing the strain (7.5% < ε_x_ < 20%), no obvious shift in *λ*_max_ was observed due to the closed distance between neighboring PS@SiO_2_ nanospheres. This color changing mechanism can be explained by Equation (3), as depicted in Figure 3k:

(3) $$\lambda =2dn_{\mathrm{eff}}$$

where *d* is the lattice spacing. During stretching, the thickness in the vertical direction is decreased due to the overall volume of the PE remaining constant. In this case, the horizontal distance between the adjacent microspheres increases while the vertical distance decreases from *d*_0_ to *d*_1_. Therefore, *λ* decreases with stretching according to Equation (3), further confirming the above experimental results. In addition, the optical response of the PEs generated by the tensile strain was kept constant and the reflection peaks remained stable at 630 nm and 619 nm during 100 tensile cycles at 10% strain, indicating its superior reproducibility and reversibility (Figure 3l).

According to Bragg’s Law, the reflection peak positions of PC films with a close-packed FCC structure can be theoretically calculated using Equations (4) and (5)

(4) $$\lambda_{\max}=2dn_{\mathrm{eff}}={\left(\frac{8}{3}\right)}^{\frac{1}{2}}Dn_{\mathrm{eff}}$$

(5) $$n_{\mathrm{eff}}={\left(n_{\mathrm{air}}{}^{2}f_{\mathrm{air}}+n_{\mathrm{nanosphere}}{}^{2}f_{\mathrm{nanosphere}}\right)}^{\frac{1}{2}}$$

where *λ*_max_ is the maximum reflection wavelength; *d* is the (111) plane spacing; D is the diameter of the nanospheres; *n*_eff_ refers to the effective refractive index; *f*_air_ = 0.26 and *f*_nanosphere_ = 0.74 are the volume fractions of the air and nanosphere, respectively; *n*_air_ = 1, *n*_PS_ ≈ 1.59, and *n*_PS@SiO2_ are the refractive indexes of the air, PS, and PS@SiO_2_, respectively. The value of *n*_PS@SiO2_ was calculated using Equation (6)

(6) $$n_{\mathrm{PS}@\mathrm{SiO}2}=\frac{V_{\mathrm{PS}}}{V_{\mathrm{PS}@\mathrm{SiO}2}}n_{\mathrm{PS}}+\frac{V_{\mathrm{SiO}2}}{V_{\mathrm{PS}@\mathrm{SiO}2}}n_{\mathrm{SiO}2}$$

where *V*_PS_ and *V*_SiO2_ are the volumes of the PS nanospheres and the SiO_2_ shell, respectively; *V*_PS@SiO2_ is the total volume of the PS@SiO_2_ nanospheres; *n*_SiO2_ ≈ 1.46 is the refractive index of SiO_2_. Here, the sizes of the blue, green, orange, red, and infrared PS@SiO_2_ nanospheres were 190.53, 214.61, 226.26, 229.73 and 282.84 nm, respectively, while the sizes of the violet, green, and orange PS nanospheres were 170.71, 208.12, and 228.46 nm, respectively (with thicknesses of the SiO_2_ shells of 9.9, 3.25, 9.7, 10.8 and 23.7 nm, respectively), as shown in Tables S1 and S2. Hence, the values of the blue, green, orange, red and infrared *n*_PS@SiO2_ were 1.55, 1.59, 1.56, 1.55, and 1.55, respectively, according to Equation (6). The calculated values of the corresponding reflection peak positions of the PC films were roughly in agreement with the observed peak positions (Figures S1–S7), as shown in Tables S3 and S4.

The PEs were prepared by infiltrating PDMS precursors into the interstices of the PS@SiO_2_ opal template. Such infiltration increases the lattice spacing of the close-packed opal template, forming a loosely packed hexagonal structure (Figure 2e). However, considering that the interparticle distance is still small after infiltration, the color shift capability is limited when adjacent nanospheres are brought closer under upon larger strain (when ε > 10%). Therefore, compared with the visible light ranges, the color shift of the PEs upon stretching is relatively small in this work. The strain required for the color change is only about 10%.

As shown in Figure 3m, the tensile stress of the PDMS film without PS@SiO_2_ colloidal crystals was 4.55 MPa, which was lower than that of the corresponding structural color film, indicating that the PS@SiO_2_ colloidal crystals also contributed to the increase in mechanical stress of the film. The angle dependence of the structural colors was demonstrated by the yellow PS@SiO_2_ PC and orange PE when the incident angle was varied from 0° to 60°. As shown in Figure 3n, 3o, the reflection wavelengths of the yellow PS@SiO_2_ PC and the orange PE gradually decreased from 567 nm to 514 nm and 598 nm to 539 nm, respectively, demonstrating their angle dependence. The same phenomenon can be observed in other PEs.

### 3.3. Patterning of the PEs

**Figure 4:**
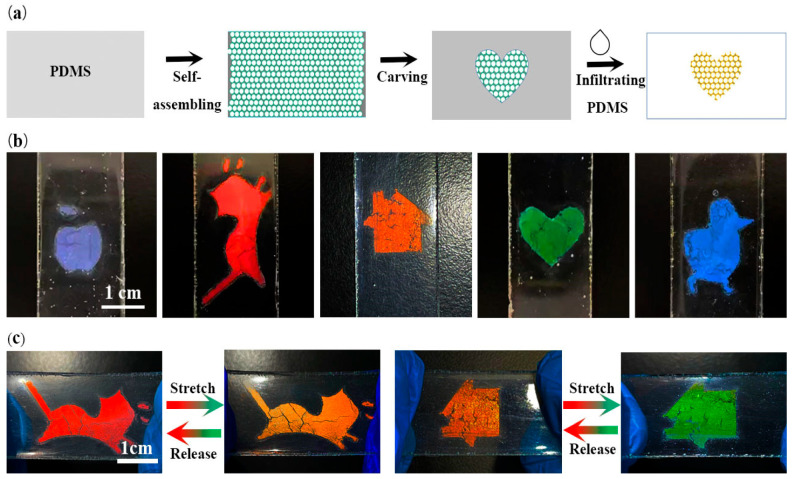
(**a**) Schematic illustration showing the fabrication process of the patterning of PE. (**b**) Photographs of PEs with different patterns and colors. (**c**) Photographs showing the color-shifting ability of the patterned PEs during stretching and releasing.

Patterned PCs play an important role in many fields, especially in anti-counterfeiting and intelligent labels [48,49,50,51]. In this work, we took a useful and easy patterning method to prepare the patterned PEs as schematically shown in Figure 4a. To achieve this, PS@SiO_2_ PCs assembled on the PDMS were carved into various patterns on demand, followed by the infiltration and curing of another PDMS layer as described earlier. Apparently, this new method offers a feasible way to prepares a variety of patterned PCs. As shown in Figure 4b, a series of PEs with different patterned were successfully prepared using the mask method (see the Materials and Methods for details). Obviously, these obtained PEs including the “apple”, “cat”, “house”, “heart” and “duck” patterns have very distinct structural color with apparent boundaries. Importantly, all of them also show excellent mechanochromic performance. As shown in Figure 4c, the red “cat” patterns of PE shifted to orange and the orange “house” pattern turned into a green “house” pattern upon stretching. Once the tensile strain was released, their structural color of PEs can be restored to the initial states, showing their outstanding color changing ability and stability.

### 3.4. Applications of the PEs as a Visualized Sensor

**Figure 5:**
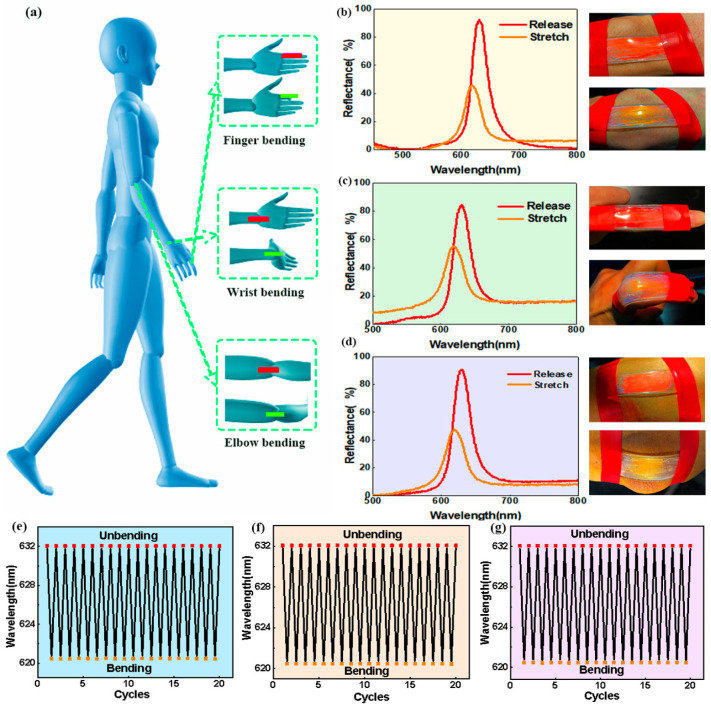
(**a**) Schematic illustration showing the application of the PE films as visualized sensors to monitor the movement states of human joints in real time; (**b**) wrist bending; (**c**) finger bending; (**d**) elbow bending; (**e**–**g**) Reflection wavelength changes of the PEs in monitoring wrist, finger, and elbow joint movements during 20 repeated cycles.

In consideration of their excellent mechanochromism, flexibility, and environmental stability, these PEs can be applied as visualized wearable devices for monitoring human motion states. When attached onto a human joint, the deformation of these PEs themselves can reflect the magnitude and frequency of the motion states in specific areas of the skin, which is crucial for human motion monitoring. In this case, the color change of a PE can visually reflect the motion state of the human joint, which can be directly perceived by human eyes and quantified by optical spectroscopy (Figure 5a). As a proof-of-concept, PEs were directly attached onto wrist, finger and elbow to monitor the motions of human joints as visualized wearable devices to demonstrate their application potential as advanced wearable interactive devices, as can be seen in Figure 5b–d. 

Taking the wrist motion as an example, it can be clearly seen that when the wrist of the human began to bend, the color changes of the PE switched from red to orange. This phenomenon was further confirmed by the reflectance spectra, with the reflection peak shifting from 632 to 618 nm (Figure 5b). Moreover, when the wrist of the human was returned to the straight position, the structural color of the PE was restored to its original state correspondingly. In addition, the structural colors and optical signal response of the PEs remained constant, even undergoing continuous wrist bending, showing excellent stability and durability in repeated motion tracking (Figure 5e). Similarly, stable optical signal responses of the PEs were also observed in other joint motion monitoring procedures, as shown in Figure 5c,d,f,g. When monitoring human motions via the PEs, their color changes were not affected by the angle variations in most cases because of the fixed observation angle.

Based on the above results, the PEs have the capability to detect movements of the human body as visualized devices. In comparison with other kinds of sensors, such PEs are able to intuitively indicate the motions of human joints through the optical signal response, which brings great application potential in human–machine interaction and health monitoring.

## 4. Conclusions

To sum up, we successfully developed a series of novel mechanochromic PEs with brilliant structural colors and stable optical responses, and demonstrated their application in the field of human motion monitoring. Notably, these PEs with a sandwich structure were obtained by embedding PS@SiO_2_ PCs within a PDMS elastomer. Due to the existence of a refractive index difference and sandwich structure, the PEs show not only brilliant structural colors, but also outstanding structural integrity. Attractively, they have admirable mechanochromic properties via lattice spacing regulation, and their optical signal response performances remain unaffected, even when suffering from 100 stretching–releasing cycles, revealing excellent reliability, durability, and stability. Furthermore, a series of PEs with colorful patterned were effectively designed and prepared using a facile mask method. Based on the above merits, we have demonstrated their versatile abilities as visualized devices for monitoring human joint motions (i.e., wrist, elbow, and finger bending). We believe that PEs will have a great application prospects in the intelligent wearable device, photonic skin, human–machine interaction, health monitoring, and many other sensing fields.

## Data Availability

Not applicable.

## Supplementary Materials

The following supporting information can be downloaded at: https://www.mdpi.com/article/10.3390/polym15122635/s1, Figure S1: (a,b) Top-view SEM images of the Violet PS PCs with different magnifications. (c) Reflectance spectrum of the PS PCs; Figure S2: (a,b) Top-view SEM images of the Red PS@SiO_2_ PCs with different magnifications. (c) Reflectance spectrum of the PS@SiO_2_ PCs; Figure S3: (a,b) Top-view SEM images of the Orange PS@SiO_2_ PCs with different magnifications. (c) Reflectance spectrum of the PS@SiO_2_ PCs; Figure S4: (a,b) Top-view SEM images of the Yellow PS@SiO_2_ PCs with different magnifications. (c) Reflectance spectrum of the PS@SiO_2_ PCs; Figure S5: (a,b) Top-view SEM images of the Green PS@SiO_2_ PCs with different magnifications. (c) Reflectance spectrum of the PS@SiO_2_ PCs; Figure S6: (a,b) Top-view SEM images of the Orange PS PCs with different magnifications. (c) Reflectance spectrum of the PS PCs; Figure S7: (a,b) Top-view SEM images of the Green PS PCs with different magnifications. (c) Reflectance spectrum of the PS PCs; Figure S8: (a) Reflectance spectra of the PE with orange structural color under various strains. (b) The position of reflection peak (λ_max_) as a function of strain; Table S1: Zeta-Sizer of three kinds of cross-linked PS emulsion; Table S2: Zeta-Sizer of five kinds of cross-linked PS@SiO_2_ emulsion; Table S3: The calculated and measured value of the reflection peak positions λ_max_ of the corresponding PS PC films; Table S4: The calculated and measured value of the reflection peak positions λ_max_ of the corresponding PS@SiO_2_ PC films.
