# Supplementary material for: Chameleon-Inspired Mechanochromic Photonic Elastomer with Brilliant Structural Color and Stable Optical Response for Human Motion Visualization
Source: Polymers (Basel). 2023 Jun 9;15(12):2635. doi: 10.3390/polym15122635 (PMC10303983; doi:10.3390/polym15122635)
Supplement: Supplementary file 1 [file polymers-15-02635-s001.zip › polymers-2376297-supplementary.pdf]

Supplementary Materials

Chameleon-Inspired Mechanochromic Photonic Elastomer with Brilliant Structural Color and Stable Optical Response for Human Motion Visualization

Yanbo Zhao †, Kai Zhao †,*, Zhumin Yu and Changqing Ye *

School of Materials Science and Engineering, Suzhou University of Science and Technology, Suzhou 215009, China; zyb1763001288@163.com (Y.Z.); yuzhumin1998@163.com (Z.Y.)
*** Correspondence: zhaokai@usts.edu.cn (K.Z.); yechangqing@mail.usts.edu.cn (C.Y.)**
† These authors contributed equally to this work.

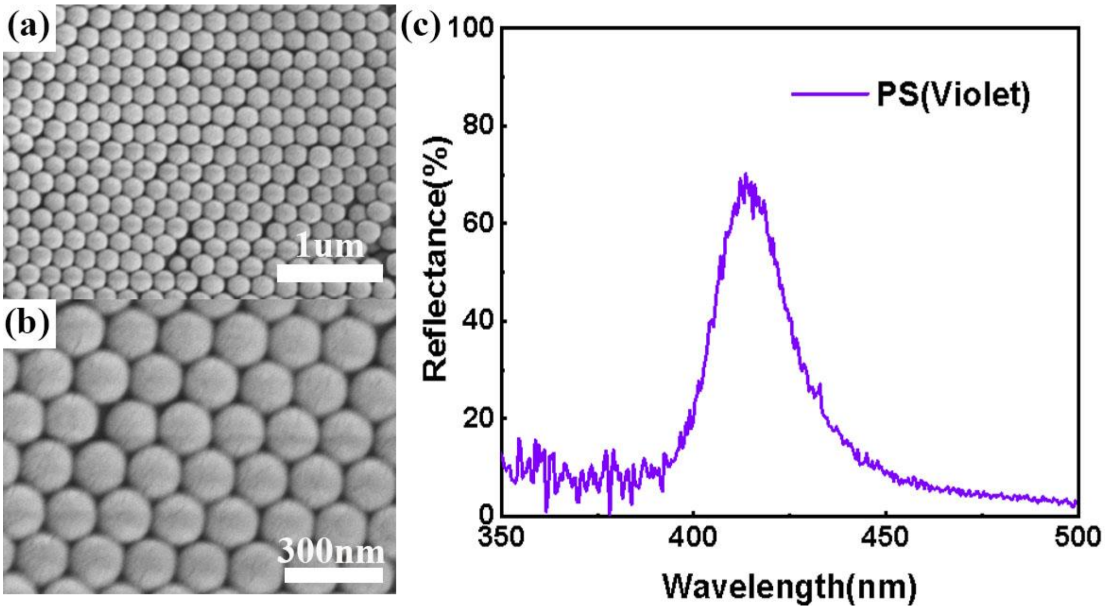

Figure S1. (a-b) Top-view SEM images of the Violet PS PCs with different magnifications. (c) Reflectance spectrum of the PS PCs.

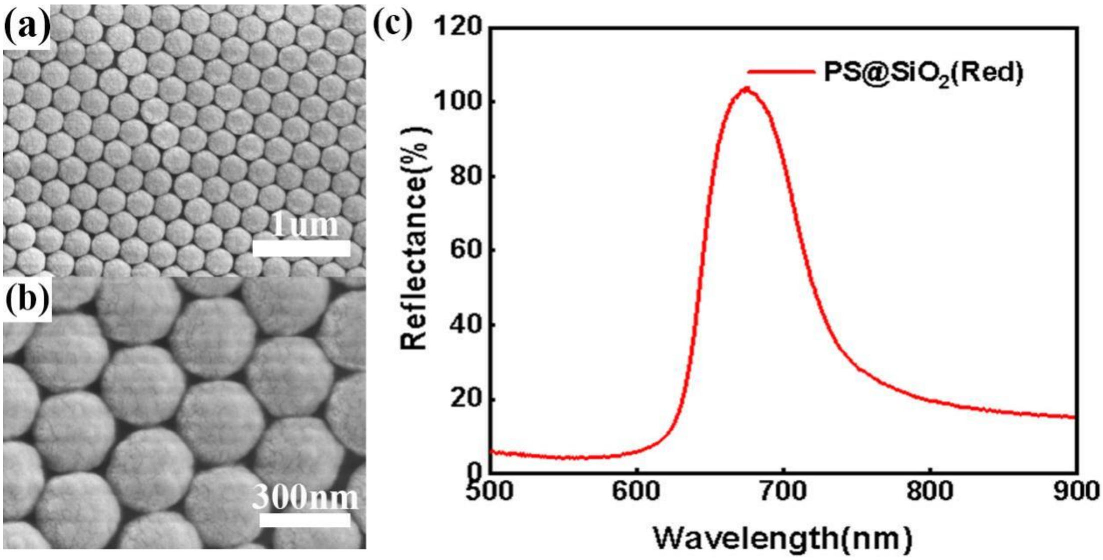

Figure S2. (a-b) Top-view SEM images of the Red PS@SiO_2 PCs with different magnifications. (c) Reflectance spectrum of the PS@SiO_2 PCs.

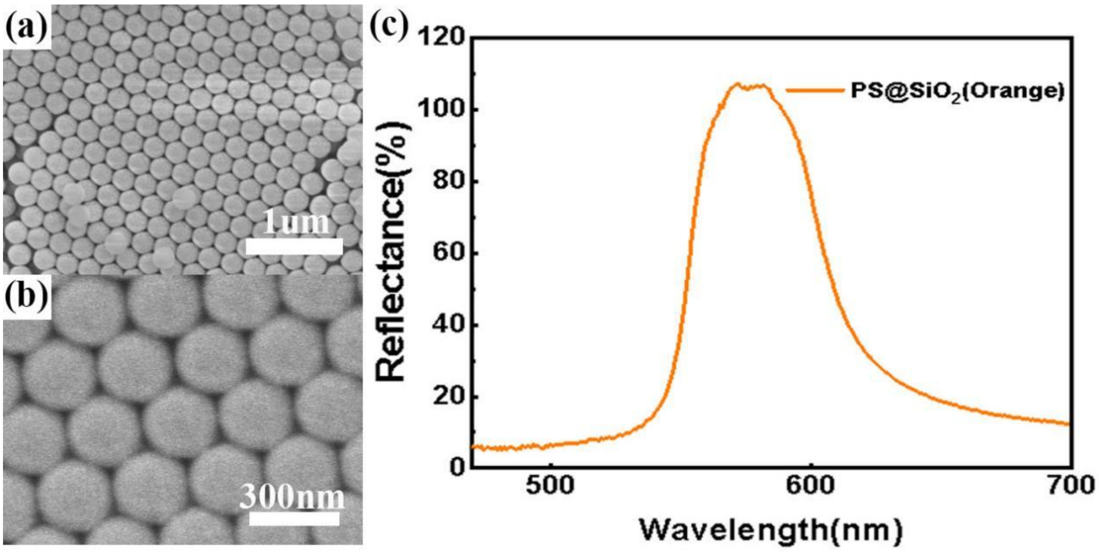

Figure S3. (a-b) Top-view SEM images of the Orange PS@SiO_2 PCs with different magnifications. (c) Reflectance spectrum of the PS@SiO_2 PCs.

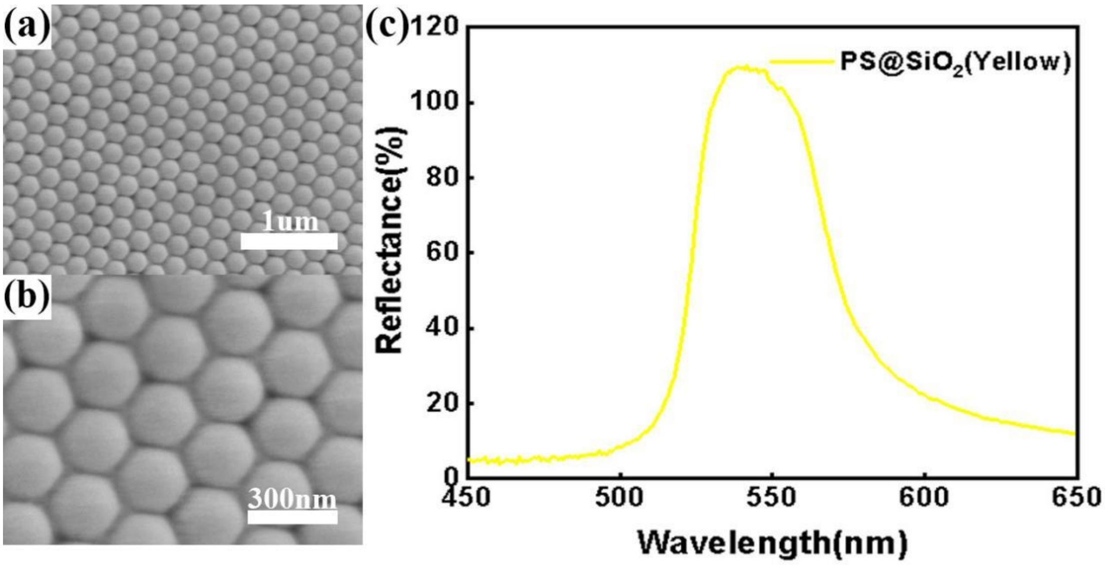

Figure S4. (a-b) Top-view SEM images of the Yellow PS@SiO_2 PCs with different magnifications. (c) Reflectance spectrum of the PS@SiO_2 PCs.

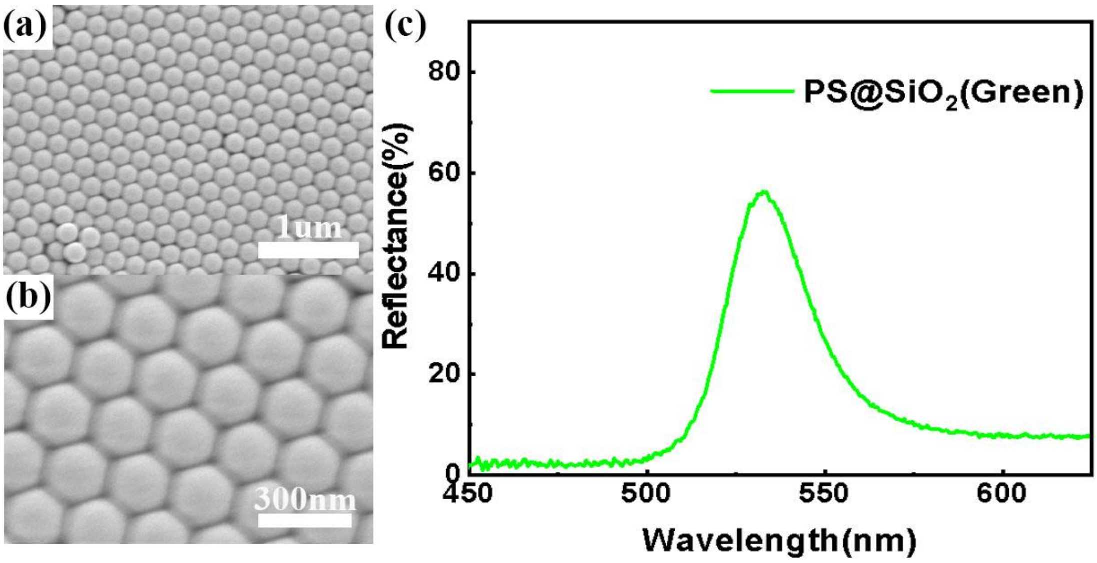

Figure S5. (a-b) Top-view SEM images of the Green PS@SiO_2 PCs with different magnifications. (c) Reflectance spectrum of the PS@SiO_2 PCs.

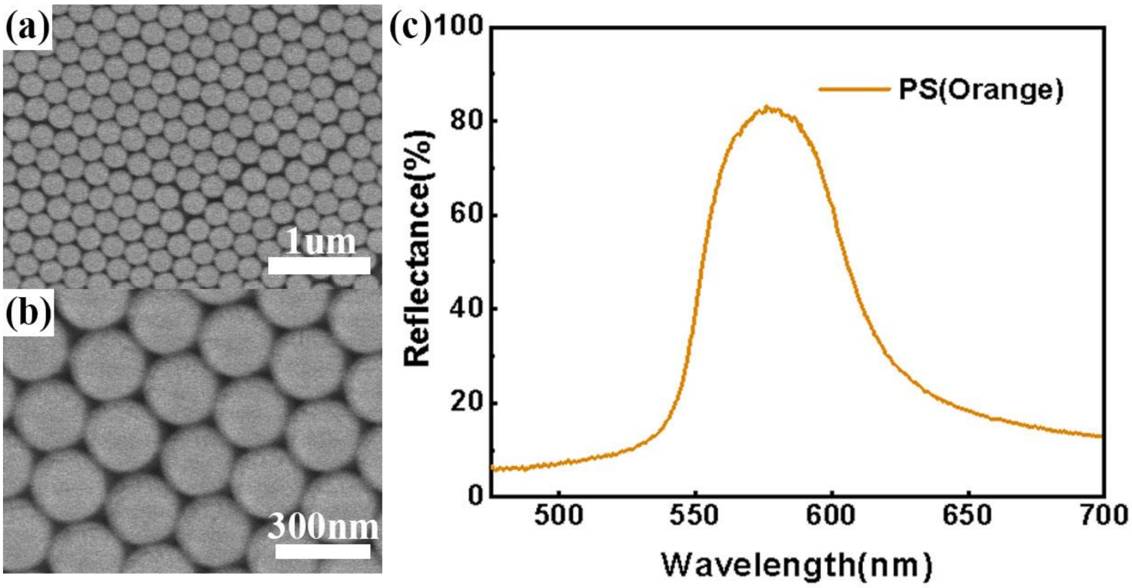

Figure S6. (a-b) Top-view SEM images of the Orange PS PCs with different magnifications. (c) Reflectance spectrum of the PS PCs.

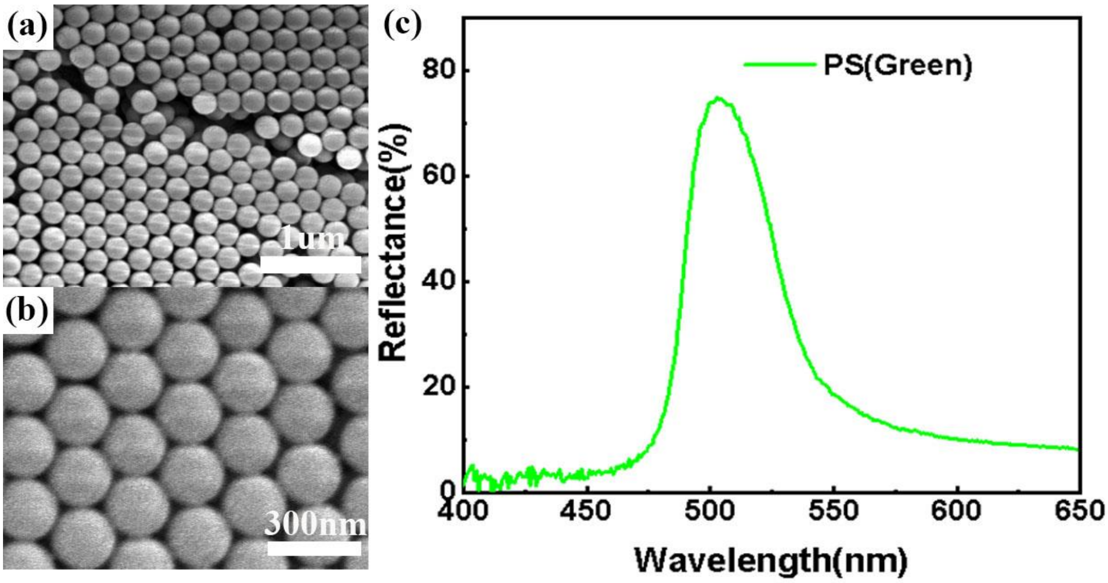

Figure S7. (a-b) Top-view SEM images of the Green PS PCs with different magnifications. (c) Reflectance spectrum of the PS PCs.

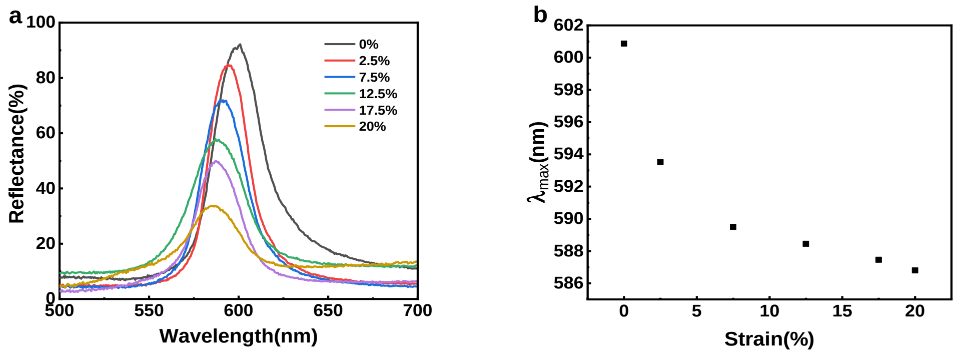

Figure S8. (a) Reflectance spectra of the PE with orange structural color under various strains. (b) The position of reflection peak (λ_{max}) as a function of strain.

Table S1. Zeta-Sizer of three kinds of cross-linked PS emulsion.

Sample	Size（nm）[a]	PDI	Zeta potential(mV)
(a) Orange	235.46	0.042	-43.1
(b) Green	208.12	0.038	-45.1
(c) Violet	170.71	0.015	-48.4

Table S2. Zeta-Sizer of five kinds of cross-linked PS@SiO_2 emulsion.

Sample	Size（nm）[a]	PDI	Zeta potential(mV)
(a) Infrared	282.84	0.009	-11.3
(b) Red	229.73	0.044	-11.9
(c) Orange	226.26	0.018	-8.67
(d) Green	208.61	0.058	-12.5
(e) Blue	190.53	0.009	-8.43

Table S3. The calculated and measured value of the reflection peak positions λmax of the corresponding PS PC films.

	Calculated value/nm	Measured value/nm
Orange PS	544.5	578.7
Green PS	495.8	504.4
Violet PS	406.9	413.5

Table S4. The calculated and measured value of the reflection peak positions λmax of the corresponding $PS@SiO_2$ PC films.

	**Calculated value/nm**	**Measured value/nm**
Infrared $PS@SiO_2$	665.1	674.2
Red $PS@SiO_2$	535.5	574.5
Orange $PS@SiO_2$	530.4	543.1
Green $PS@SiO_2$	514.1	532.5
Blue $PS@SiO_2$	440.1	441.2
